# Biodegradation Ability and Catabolic Genes of Petroleum-Degrading *Sphingomonas koreensis* Strain ASU-06 Isolated from Egyptian Oily Soil

**DOI:** 10.1155/2014/127674

**Published:** 2014-08-10

**Authors:** Abd El-Latif Hesham, Asmaa M. M. Mawad, Yasser M. Mostafa, Ahmed Shoreit

**Affiliations:** ^1^Genetics Department, Faculty of Agriculture, Assiut University, Assiut 71516, Egypt; ^2^Biology Department, Faculty of Science, King Khalid University, Abha 61321, Saudi Arabia; ^3^Botany and Microbiology Department, Faculty of Science, Assiut University, Assiut 71516, Egypt; ^4^Egyptian Petroleum Research Institute, Nasr City, Cairo 11727, Egypt

## Abstract

Polycyclic aromatic hydrocarbons (PAHs) are serious pollutants and health hazards. In this study, 15 PAHs-degrading bacteria were isolated from Egyptian oily soil. Among them, one Gram-negative strain (ASU-06) was selected and biodegradation ability and initial catabolic genes of petroleum compounds were investigated. Comparison of 16S rRNA gene sequence of strain ASU-06 to published sequences in GenBank database as well as phylogenetic analysis identified ASU-06 as* Sphingomonas koreensis*. Strain ASU-06 degraded 100, 99, 98, and 92.7% of 100 mg/L naphthalene, phenanthrene, anthracene, and pyrene within 15 days, respectively. When these PAHs present in a mixed form, the enhancement phenomenon appeared, particularly in the degradation of pyrene, whereas the degradation rate was 98.6% within the period. This is the first report showing the degradation of different PAHs by this species. PCR experiments with specific primers for catabolic genes* alkB, alkB1, nahAc, C12O*, and* C23O* suggested that ASU-06 might possess genes for aliphatic and PAHs degradation, while* PAH-RHD*α*GP* gene was not detected. Production of biosurfactants and increasing cell-surface hydrophobicity were investigated. GC/MS analysis of intermediate metabolites of studied PAHs concluded that this strain utilized these compounds via two main pathways, and phthalate was the major constant product that appeared in each day of the degradation period.

## 1. Introduction

Polycyclic aromatic hydrocarbons (PAHs) are a group of hydrophobic organic compounds composed of two or more fused aromatic rings in their chemical structure [1]. PAHs are released into the environment from the incomplete combustion of fossil fuels and organic matter, the accidental spilling of processed hydrocarbons and oils, run-off from asphalt pavements, coal liquefaction, and gasification, and natural geological processes [2]. Due to their toxic, carcinogenic, and mutagenic properties, PAHs are of environmental and human concern, and 16 PAHs have been listed by the US Environmental Protection Agency as priority contaminants in ecosystems [3].

Microbial degradation is the most dominant and significant process for removing PAHs from the environment. Many microorganisms capable of metabolizing PAHs were isolated including bacteria [4], yeasts [5], fungi [6], and algae [7]. Most of the bacteria isolated belong to genera* Pseudomonas*,* Burkholderia*,* Mycobacteria*,* Rhodococcus*,* Alcaligenes*,* Ralstonia*, and others [4, 8].

Usually, contaminated sites are polluted by a mixture of PAHs. Thus, for an efficient remediation process, it is important that the bacteria involved have a complete degradation pathway so that no potentially toxic degradation products accumulate [9]. Genetic analyses of PAH catabolic pathways in several PAH-degrading bacteria revealed the presence of a group of genes for complete degradation of aromatic compounds [10, 11]. PCR amplification using genes specific primers or degenerate primers has been used to detect and study the diversity of aromatic-dioxygenase genes in PAH-degrading bacterial isolates [12–14].

Although many bacteria capable of degrading PAHs have already been isolated, it is still important to screen strains that can completely and rapidly decompose PAHs under the condition of Egyptian environment. Therefore, the aim of the present study was to isolate strains capable of degrading low and high molecular weight PAHs. A strain was obtained and identified as* S. koreensis* according to morphological characteristics and 16S rRNA gene sequence analysis and its ability to degrade naphthalene, phenanthrene, anthracene, and pyrene were studied. The production of biosurfactants and increasing cell-surface hydrophobicity, the metabolites during the degradation process, and the genetics of catabolic genes in the isolated PAH-degrading bacterium were also investigated.

## 2. Materials and Methods

### 2.1. Sample Collection and Chemicals

Oil contaminated soil was collected in sterilized polyethylene bags from Oil Refinery Company in Assiut, Egypt, and stored at 4°C in the laboratory. Naphthalene, phenanthrene, anthracene, pyrene (all ≤99% purity), and mineral basal medium with trace metals were purchased from Sigma-Aldrich.

### 2.2. Enrichment, Isolation, and Evaluation of PAHs-Degrading Bacteria

Soil enrichment technique was used for the isolation of PAH-degrading bacteria as described in [5]. About 10 g oil contaminated soil sample was suspended in 90 mL mineral basal salt medium (MBS) containing (g/L) 1.0(NH_4_)_2_SO_4_, 0.8K_2_HPO_4_, 0.2KH_2_PO_4_, 0.2MgSO_4_ *·*7H_2_O, 0.1CaCl_2_ *·*2H_2_O, 0.005FeSO_4_ *·*7H_2_O, and 1 mL of trace elements, pH 7.0 ± 0.2. The medium was supplemented with one of the following PAHs: naphthalene, phenanthrene, anthracene, or pyrene at concentration of 100 mg/L as a sole source of carbon. The flasks were incubated on an orbital shaker at 150 rpm at 30°C. After 7-day incubation, an aliquot of 10% enriched cultures was transferred into another 250 mL conical flask containing 90 mL fresh autoclaved MBS medium supplemented with previously mentioned PAHs. This step was repeated five times to attain well-adapted PAH-degrading enriched bacterial consortia. Bacterial strains were isolated from MBS agar plates coated with the same PAHs as the sole carbon source. Colonies with different morphologies were individually selected. All isolates were evaluated for their PAHs-degrading abilities. One pure strain of bacteria with a high PAHs degradation rate was designated as ASU-06 and selected for further study.

### 2.3. Physiological and Biochemical Tests of Strain ASU-06

Conventional physiological and biochemical characteristics were determined using the procedures described by John and Krieg [15].

### 2.4. 16S rRNA Gene Amplification and Sequence Determination

The genomic DNA was isolated from strain ASU-06 according to the method described by Hesham [16] and the 16S rRNA gene was amplified. Amplification was carried out with universal primers: 27F (5-AGAGTTTGATCCTGGCTCAG-3) and 1492R (5-CGGCTACCTTGTTACGACTT-3) in a final volume of 50 *μ*L containing 10 mM tris-HCl (pH 8.3), 50 mM KCl, 1.5 mM MgCl_2_, each dNTP at a concentration of 0.2 mM, 1.25 IU of Taq polymerase, each primer at a concentration of 0.2 mM, and 1 *μ*L of the DNA template. PCR was performed with the following program: 5 min denaturation at 95°C, followed by 36 cycles of 1 min denaturation at 94°C, 1 min annealing at 55°C, 1.5 min extension at 72°C, and a final extension step of 7 min at 72°C. 5 *μ*L of the amplified mixture was then analyzed using 1.5% 0.5 × TBE agarose gel electrophoresis. The gel was stained with ethidium bromide, visualized under UV light, and photographed. Product of the correct size was purified and sequenced in both directions using an ABI automated sequencer.

### 2.5. Sequence Alignment and Phylogenetic Analysis

The 16S rRNA gene sequences of the isolate obtained in this study were aligned and compared with the known 16S rRNA gene sequences in Genbank database using the BLAST search at the National Center for Biotechnology Information (http://www.ncbi.nlm.nih.gov/BLAST/) to determine the closest available database sequences. To determine the taxonomic position of the isolate, a phylogenetic tree was constructed with MEGA version 4.0 using a neighbor-joining algorithm; in addition, the Jukes-Cantor distance estimation method with bootstrap analyses for 1,00 replicates was performed [17].

### 2.6. PAHs Degradation Analysis by HPLC

Pure strain was resuspended in 50 mL MBS supplemented with either naphthalene, phenanthrene, anthracene, or pyrene at concentration of 100 mg/L for each or mixed of them PAHs (25 mg/L) for each to a turbidity of 0.2 at OD (600 nm). Abiotic controls were prepared in the same way but without addition of bacteria. Samples and controls were prepared in triplicate. Flasks were incubated at 30°C with rotation at 150 rpm for 15 days. After every three days of incubation, 25 mL aliquot of each sample was extracted three times with equal volumes of ethyl acetate, according to the method described by Manohar et al. [18]. The residual PAHs were analyzed by HPLC as described by Bishnoi et al. [19].

### 2.7. Detection of PAH-Degrading Enzymes

The initial dioxygenase activity catalyzing the conversion of indole to be indigo was determined following the standard method described by a previous worker [20], while Catechol 2,3-dioxygenase activity was determined according to Ornston and Stanier [21].

### 2.8. Detection of Aliphatic and PAH-Degrading Genes by PCR

The presence of six genes including monooxygenase and dioxygenase genes in the isolated bacterial strain ASU-06 was detected based on PCR amplification. The primers for the detection of n-alkanes monooxygenase (*alkB* and* alkB1*), dioxygenase (*nahAc*), Catechol dioxygenase (*C12O* and* C23O*), and PAH-ring hydroxylating dioxygenase (*PAH-RHD* *α*) genes were listed in Table 1. PCR conditions were initial denaturation for 5 min at 95°C, 35 cycles with 40 s at 94°C, 40 s at 55°C, 60 s at 72°C, and final elongation for 7 min at 72°C for the four genes* alkB, alkB1, nahAc,* and * PAH-RHD* *α* [12–14, 22]. However, PCR was performed for Catechol 1,2-dioxygenase (*C12O*) and Catechol 2,3-dioxygenase (*C23O*) genes with initial denaturation for 5 min at 95°C, 35 cycles with 20 s at 94°C, 30 s at 63°C, and 45 s at 72°C, and final elongation for 5 min at 72°C [23]. All PCR products were separated in 1.5% agarose gel, stained with ethidium bromide, visualized under UV light, and photographed.

### 2.9. Detection Cell-Surface Properties

Production of extracellular biosurfactant by the culture was analyzed by monitoring the ability of surfactant to stabilize 1-naphthaldehyde in water emulsion as described earlier by Phale et al. [24]. An aliquot of 5 mL of sample was centrifuged at 2000 rpm, the pellet was discharge, and the supernatant was used as a source of biosurfactant(s). The total assay mixture (5 mL) contained 200 *μ*L supernatant, 3.8 mL phosphate buffer (50 mM, pH 7.5), and 1 mL of 1% of 1-naphthaldehyde in water emulsion (100 *μ*L naphthaldehyde in 10 mL double distilled water followed by 1 min sonication). The mixture was vortexed for 1 min and incubated at room temperature for 5 h. The absorbance due to stability of emulsion was measured at 660 nm against control that was prepared by using 200 *μ*L distilled H_2_O instead of the supernatant. One unit is defined as the amount of biosurfactants required to obtain an increase in absorbance of 1.0 OD unit. Affinity of cells towards various PAHs was measured by the method of Rosenberg et al. [25]. Cells were grown on PAHs harvested and washed twice with phosphate buffer (50 mM, pH 7.5) and resuspended in the same buffer to obtain a cell suspension with a final OD of 0.300 at 600 nm. The 6 mL assay mixture contained 3 mL cell suspension and 3 mL test PAHs. After 5 min of preincubation, the mixture was vortexed for 60 s and incubated for additional 15 min at room temperature. The OD of the aqueous phase was measured at 600 nm. The cell-surface hydrophobicity was expressed as percent cells transferred to hydrocarbon phase by measuring the OD of the aqueous phase before and after mixing.

### 2.10. Identification of PAHs Metabolites Using GC-MS Analysis

After growth on PAHs, contents of the flasks were extracted with three equal volumes of ethyl acetate. The aqueous fraction after extraction was acidified with concentrated HCl to pH 2 and extracted again with three equal volumes of ethyl acetate. The residual extracts were dried over anhydrous sodium sulfate and evaporated with rotatory evaporator at 40°C to 10 mL [26]. The samples were dried in vacuum and stored at −20°C until being used. The extract was then dissolved in hexane and introduced for GC-MS analysis. GC-MS analysis was performed using an HP 6890 gas chromatograph with an HP 5973 mass spectrometer system. The column was a TR-5MS (5% phenyl polysilphenylene siloxane) (30 m × 0.25 mM diameter, 0.25 uM film thickness). Helium was the carrier gas, at 1 mL/min constant flow. The column temperature was held at 70°C for 5 minutes, increased at a rate of 4°C/min to 290°C, and held for 10 minutes. To remove any remaining compounds, the analysis was finished with a ramp of 20°C/min to 320°C held for 20 minutes. The mass spectrometer was operated in electron impact (EI) mode at 70 electrons volts (EV) in the full scan mode from 85 to 450* m/z* over 6.5–85 minutes. Injector and detector temperatures were 270°C and 280°C, respectively [5, 17].

### 2.11. GenBank Accession Number

The nucleotide sequences of 16S rRNA gene of* Sphingomonas* strain ASU-06 reported in this study have been deposited in the DDBJ, EMBL, and GenBank nucleotide sequence databases under Accession Number KC420523.

## 3. Results

### 3.1. Isolation, Characterization, and Evaluation of PAHs-Degrading Bacteria

The 15 bacterial colonies were isolated from Egyptian oily soil. By visualization, these strains were varied in colony shape, color of colony, and levels of growth on solid MBS medium. Among these isolates, one Gram-negative strain ASU-06 was selected for further biodegradation studies of PAHs owing to its highest growth on all used PAHs as a sole source of carbon. Strain ASU-06 showed an ability to produce indigo (blue) colored colonies as a result of dioxygenase activity in the presence of indole as a substrate. It was also recorded positive when Catechol was introduced as a substrate and formed yellowish or brown colonies. Conventional physiological and biochemical characteristics were determined for ASU-06 using the procedures described by John and Krieg [15]; the results concluded that ASU-06 could be identified as* Sphingomonas* sp.

### 3.2. Molecular Identification and Phylogenetic Analysis Using 16S rRNA Gene Sequence

**Figure 1:**
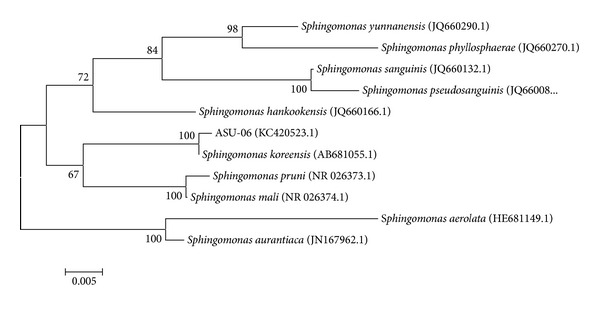
Phylogenetic analysis of 16S rRNA gene of isolate ASU-06 and other related* Sphingomonas *spp. By neighbor-joining method. Numbers at the nodes indicate bootstrap support (%) based on 100 replicates. The scale bar indicates 0.005 nucleotide substitutions per nucleotide position. GenBank accession numbers are given in parentheses.

The genomic DNA was extracted from the isolated bacterial strain ASU-06 and universal primers 27F and 1492R were used for the amplification and sequencing of the 16S rRNA gene fragment. The alignment and comparison of the 16S rRNA gene sequence of the strain ASU-06 to the published 16S rRNA gene sequences in GenBank database by BLAST search were determined. Results show that the 16S rRNA gene sequence of the isolated strain was highly homologous to* Sphingomonas koreensis*, with 100% sequence similarity. To confirm the position of the strain ASU-06 in phylogeny, a number of sequences representative of some* Sphingomonas* species were selected from Genbank database for construction of a phylogenetic tree. As shown in Figure 1, the phylogenetic tree indicated that strain ASU-06 and* S*.* koreensis* shared one cluster. Therefore, the strain ASU-06 was identified as* S. koreensis*.

### 3.3. PAHs Biodegradation Analysis Using HPLC

**Figure 2:**
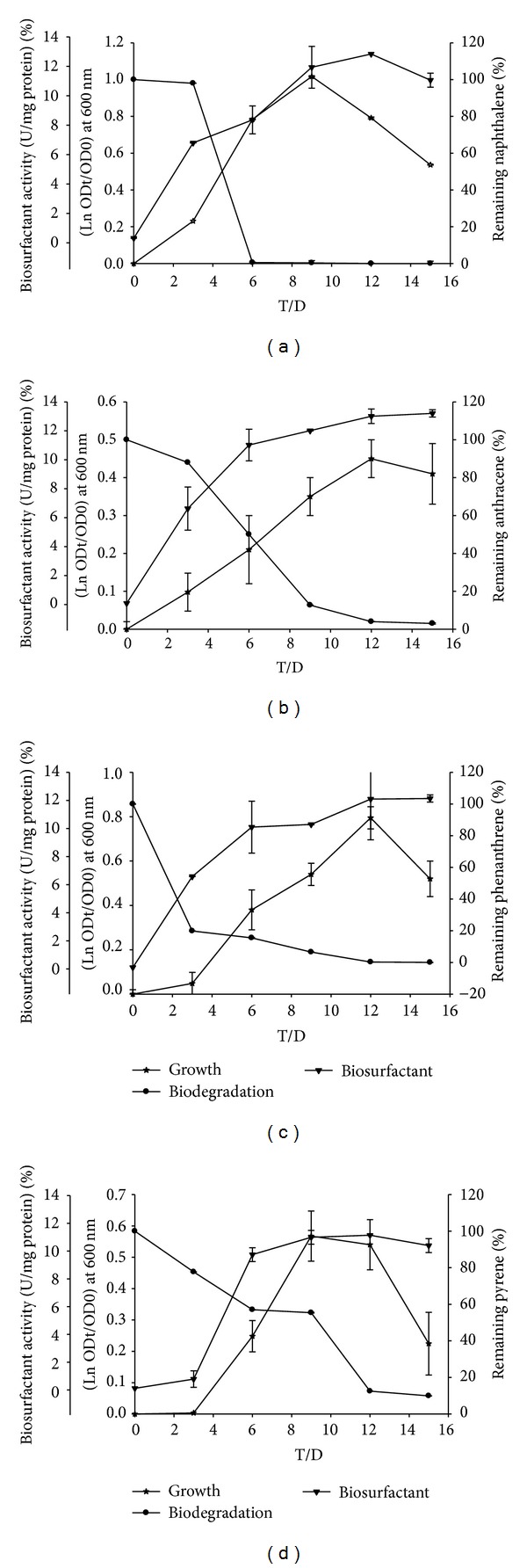
The relation between growth curve, % of PAHs degradation, and activity of biosurfactants (U/mg protien) of* S. koreensis*, growing on MBS supplemented with 100 mg/L of (a) naphthalene, (b) anthracene, (c) phenanthrene, and (d) pyrene “as single substrate.”

**Figure 3:**
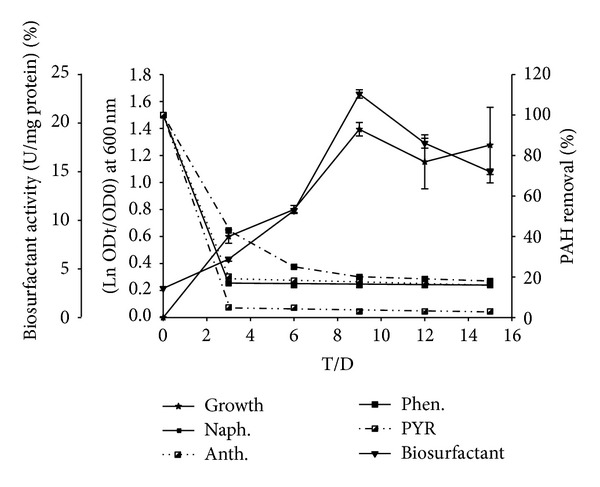
The relation between growth curve at OD 600, % of PAHs degradation, and activity of biosurfactants (U/mg protien) of* S. koreensis*, growing on MBS supplemented with 100 mg/L of four PAHs “as mixed substrate.”

Degradation of sole PAHs by* S. koreensis* strain ASU-06 in liquid cultures is shown in Figure 2.* S. koreensis* could degrade the four PAHs, naphthalene, phenanthrene, anthracene, and pyrene, at concentration of 100 mg/L for each with different capabilities of 100, 99, 98, and 92.7% after 15 days, respectively. When these PAHs present in a mixed form, the enhancement phenomenon appeared (Figure 3), particularly in degradation of pyrene, whereas the degradation rate was 98.6% within 15 days.

### 3.4. Detection of Catabolic Genes by PCR

**Figure 4:**
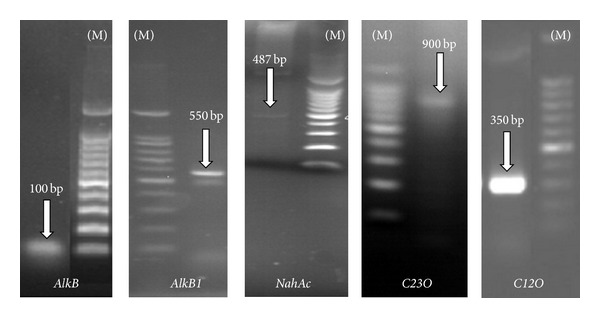
PCR products based on primers specific for the catabolic genes from the left to the right,* AlkB*,* AlkB1*,* nahAc*,* C23O*, and* C12O*, that are detected in* S. koreensis*, with 100 bp DNA ladder (M).

The catabolic genes can be used to trace the specific activities of bacterial strain. We targeted six key enzyme coding genes including monooxygenase and dioxygenase responsible for the mineralization of aliphatic and PAHs compounds. The presence of six genes in* S. koreensis* strain ASU-06 was detected based on PCR amplification. The existence of monooxygenase (*alkB* and* alkB1*), dioxygenase (*nahAc*), and Catechol dioxygenase (*C12O* and* C23O*) genes was confirmed, and the results are shown in Figure 4 and Table 2. The sizes of PCR products for monooxygenases* alkB* and* alkB1* were approximately 100 bp and 550 bp, respectively, while for* nahAc*,* C12O*, and* C23O*, they were 487, 350, and 900 bp, respectively. Finally, no signal for* PAH-RHD* *α* gene was detected.

### 3.5. Biosurfactant Production and Cell-Surface Hydrophobicity

Production of extracellular biosurfactant by the culture and affinity of cells towards various PAHs were investigated. The emulsification activity was increased gradually from lag to reach the maximum values at exponential phase (Figures 2 and 3). The values of percentage of cell-surface hydrophobicity (CSH) toward naphthalene, phenanthrene, anthracene, pyrene, and mixed PAHs were 37, 57, 63, 37, and 65%, respectively. The increasing of cell-surface hydrophobicity facilitated the direct contact between cells and the substrate particles that will stimulate the growth of organism.

### 3.6. Detection of PAH Metabolic Products Produced by GC-MS Analysis

All detected metabolites, retention times, and fragmentation patterns were summarized in (Table 3). It was noted that phthalate was the major constant product that appeared in each day of the degradation period. The other metabolic products were different and changed every three days. During degradation of naphthalene and phenanthrene, the presence of cinnamate, salicylate, and phthalate indicates that this strain has two major pathways for naphthalene degradation. Detecting of 1-hydroxyl or dihydroxyl PAHs derivatives is confirming the activity of* nahAc* gene. Appearance of to cis,cis-dihydroxyl muconate during both phenanthrene and pyrene degradation confirms the activity of* C12O* enzyme, and also it could be concluded that the ortho pathway is major for these carbon sources degradations.

The percentage of phthalate increased gradually from 17.89% to 57.25 at the 12th day and this percentage was decreased due to consumption to form other metabolites during pyrene assimilation.* S. koreensis* was tested to grow on phthalate as a sole source of energy on MBS agar medium, and growth was obtained after 24 hours. According to the detected metabolic products, it could be supposed the expected metabolic pathway of pyrene degradation as an example of four rings PAHs by* S. koreensis*.

## 4. Discussion

PAHs are serious pollutants and health hazards, and they occur as complex mixtures, including low and high molecular weight; therefore, degradation of PAHs in the environment is becoming more necessary and interesting. Low molecular weight is more easily degraded, while high molecular weight is more recalcitrant and requires specific microorganisms to perform the degradation. Construction of consortia by mixing several known PAH degraders has failed to maximize cooperation among different species using synthetic consortia [27]. Therefore, the powerful way to obtain the promising strain that could utilize PAHs as the sole carbon source is the enrichment techniques [5]. Since only a few reports on the microbial metabolism of PAHs with four or more aromatic rings were published, they too mainly focused on cometabolic transformations [28]. Therefore, in this study, 15 isolates were obtained from Egyptian oily soil using an enriched technique. From these isolates, one Gram-negative strain ASU-06 was chosen based on its utilization of naphthalene, phenanthrene, anthracene, and pyrene as a sole carbon source and the ability for dioxygenase activities. Mrozik et al. [29] reported that most of PAHs degradable bacteria are Gram-negative. Results of conventional characteristics concluded that ASU-06 could be identified as* Sphingomonas* sp. The alignment and comparison of the 16S rRNA gene sequence of the strain ASU-06 to the published 16S rRNA gene sequences in GenBank database as well as phylogenetic analysis confirmed the taxonomic position of the strain as* Sphingomonas koreensis* (Figure 1). Species of the genus* Sphingomonas* were isolated from different samples and have shown degradation capability toward different PAHs [30]. Several PAH-degrading bacterial strains isolated from mangrove sediments belonging to the genera of* Sphingomonas*,* Mycobacterium*,* Paracoccus,* and* Rhodococcus* showed different potential in degrading mixed PAHs [31]. Results reported by Uyttebroek et al. [32] demonstrated that* Mycobacterium* strains had higher rate in degrading phenanthrene than* Sphingomonas* strains. Our results in Figure 2 showed the degradation rates of sole PAH by* S*.* koreensis* strain ASU-06 in liquid cultures. This bacterium almost completely degraded 100 mg/L of naphthalene, phenanthrene, and anthracene, while the degradation rate for pyrene was 92.7% during 15 days. On the other hand, when the 4 PAHs were simultaneously presented, degradation rate was enhanced from 92.7% to 98.6% for pyrene (Figure 3). Similar phenomenon has been observed by other researchers in different microorganisms. Yuan et al. [33] reported that the degradation efficiency using isolated bacteria is improved when acenaphthene, fluorene, phenanthrene, anthracene, and pyrene are present together compared to PAHs present individually. In our previous work [5] using isolated yeast, we found that naphthalene was promoting the degradation of high molecular weight more than other components.

Degradation of aliphatic and PAHs was mostly carried out by the mono- and dioxygenases produced by bacteria. Therefore, the presence of six key enzyme coding genes including both monooxygenase and dioxygenase in* S. koreensis* strain ASU-06 was investigated. The existence of monooxygenase (*alkB *and* alkB1*), dioxygenase (*nahAc*), and Catechol dioxygenase (*C12O* and* C23O*) genes was confirmed. A possible reason may be because these detected genes are highly conserved among different Gram-negative bacteria. However, no signal for* PAH-RHD* *α* gene was detected since it is conserved among Gram-positive bacteria [12]. The naphthalene dioxygenase (*nahAc*) gene is of particular interest as an indicator for PAHs degradation because the enzyme encoded by this gene not only degrades naphthalene, but also mediates degradation of other PAHs compounds [31, 34].* C12O* and* C23O* dioxygenases play a key role in the metabolism of aromatic rings by the bacteria because they are responsible for cleavage aromatic C–C bond at ortho or meta position [35]. On the other hand, the presence of catabolic genes (*alkB* and* alkB1*) should be investigated because short-chain n-alkanes are directly toxic, acting as solvents for cellular fats and membranes [36]. Our results demonstrated that the five genes were present in the* S. koreensis* strain ASU-06, suggesting that this bacterium played an important role in degradation of aliphatic and PAHs and could be recommended for petroleum compounds bioremediation in the environment.

Recent studies have suggested that microorganisms involved in the degradation of hydrophobic compounds like PAHs and alkanes possess special physiological properties [37]. To investigate the effects of physiological properties on PAHs degradation, the emulsification activities and cell-surface hydrophobicity of* S. koreensis* strain ASU-06 were detected. The emulsification activity was increased gradually from lag to reach the maximum values at exponential phase (Figures 2 and 3). Although both phenanthrene and anthracene consist of three rings, the solubility of the phenanthrene 1290 *μ*g/L is more than that of the anthracene 73 *μ*g/L [1]. However, biosurfactant production by bacteria increased toward anthracene rather than phenanthrene [38, 39]. This finding was assured by Pei et al. [40] who demonstrated that biosurfactant (rhamnolipid) and cell-surface hydrophobicity production by* Sphingomonas* sp. increased the solubility of phenanthrene and subsequently enhanced the utilization/uptake of carbon source. The values of % CSH toward naphthalene, phenanthrene, anthracene, pyrene, and mixed PAHs were 37, 57, 63, 37, and 65%, respectively. The increase in CSH facilitated the direct contact between cells and the substrate particles that will stimulate the growth of organism. The production of biosurfactants and increasing CSH are two possible strategies used by microorganisms to improve the transportation of hydrophobic hydrocarbon substrates to cells [39].

GC/MS analysis of intermediate metabolites of two, three, and four rings PAHs showed that phthalate was the major constant product that appeared in each day of degradation period. The metabolic products were different and changed every three days. The percentage of phthalate increased gradually from 17.89 to 57.25% at the 12th day and this percentage was decreased due to consumption to form other metabolites.* S. koreensis* was tested to grow on phthalate as a sole source of energy on MBS agar medium; the heavy growth was obtained after 24 hours. Zhong et al. [41] detected only two metabolites (monohydroxypyrene and pyrene diol) from pyrene degradation by mixed culture of* Sphingomonas* and* Mycobacterium,* while, when* Sphingomonas* was used as pure culture for pyrene degradation, no metabolites could be detected.

The accumulation of monohydroxypyrene at the first step of degradation indicates that the strain added one atom of oxygen via monooxygenase enzyme to form 1-hydyoxypyrene and then added another one of O_2_ via pyrene dioxygenase to form either 4,5-dihydroxypyrene or 1-methoxy-2-hydroxypyrene. Detection of phenanthrene dicarboxylic acid in the present study supports the presence of an orthocleavage pathway of 1,2- and 4,5-dihydroxypyrene [42]. Phenanthrene-4,5-dicarboxylic then converted to 1-hydroxy-2-naphthoic acid. Two common metabolic pathways for 1-hydroxy-2-naphthoic acid are (a) formation of 2-carboxy benzal pyruvate under the catalysis of 1-hydroxy-2-naphthoate dioxygenase [43] and (b) dihydroxynaphthalene under the catalysis of salicylate-1-hydroxylase [44]. In the present study, 2-carboxy benzal pyruvate was found but there were no traces of dihydroxynaphthalene. A number of Gram-negative and Gram-positive bacteria in the genera of Pseudomonades,* Nocardioides,* and* Mycobacterium* are known to have proteins or genes of 1-hydroxy-2-naphthoic acid dioxygenase [42, 43]. These results well agreed with the results of Kim et al. [42] and Seo et al. [43]. Phthalate, terephthalate, and 4-hydroxybenzoate were found to be degraded via protocatechuate orthocleavage in* Ralstonia jostii* RHA1 [45]. The Sphingomonads were known to possess broad PAHs metabolic capabilities [46], and they were involved in degradation of various recalcitrant organic pollutants, such as biphenyl [47] and benzo[a]pyrene [28]. Furthermore, it seems likely that the degradation of individual PAH compounds by the isolated bacteria proceeds via independent pathways [10]. From the metabolic pathway of* S. koreensis,* it could be concluded that this strain utilized 100 mg/L of naphthalene (two rings), phenanthrene (three rings), and pyrene (four rings) via two main pathways.

In summary, the present study reports the isolation, identification, and characterization of a very efficient bacterium species capable of degrading low and high molecular weight (LMW and HMW) PAHs as a sole source of carbon and energy. The bacterium strain ASU-06 was identified based on 16S rRNA gene sequence phylogeny and comparison of this gene sequence with sequence in 16S rRNA gene database available in GenBank as* S. koreensis*. This bacterium almost completely removed 100 mg/L of LMW-PAHs, while the degradation rate for pyrene as a HMW-PAH was 92.7% during 15 days. When the 4 PAHs were simultaneously present, degradation rate was enhanced for pyrene from 92.7% to 98.6%. The existence of catabolic genes including monooxygenase (*alkB* and* alkB1*), dioxygenase (*nahAc*), and Catechol dioxygenase (*C12O* and* C23O*) genes responsible for aliphatic and polyaromatic hydrocarbons degradation was confirmed, while no signal for* PAH-RHD*α*GP* gene for Gram-positive strains was detected. GC/MS analysis of intermediate metabolites of LMW and HMW-PAHs concluded that this strain utilized 100 mg/L of naphthalene (two rings), phenanthrene (three rings), and pyrene (four rings) via two main pathways and phthalate was the major constant product that appeared in each day of degradation period. Our results suggest that this bacterium played an important role in degradation of aliphatic and polyaromatic hydrocarbons and could be recommended for petroleum compounds bioremediation in the environment.

## Figures and Tables

**Table 1:** List of primers used for detection of mono- and dioxygenase genes.

Primer	Sequence (5′ to 3′)	Expected size (bp)	Reference
alkBF alkBR	5′-AACTACMTCGARCAYTACGG-3′ 5′-TGAMGATGTGGTYRCTGTTCC-3′	100	Powell et al. [22]
AlkB1F AlkB1R	5′-TACGGGCACTTCGCGATTGA-3′ 5′-CGCCCAGTTCGAMACGATGTG-3′	550	Kloos et al. [13]
nahAc F nahAc R	5′-TGGCGATGAAGAACTTTTCC-3′ 5′-AACGTACGCTGAACCGAGTC-3′	487	Laurie and Jones [14]
PAH-RHD-GPf PAH-RHGPr	5′-CGG CGC CGA CAA YTT YGT NGG-3′ 5′-GGG GAA CAC GGT GCC RTG DAT RAA-3′	292	Cébron et al. [12]
C12OF C12OR	5′-GCCAACGTCGACGTCTGGCAGCA-3′ 5′-CGCCTTCAAAGTTGATCTGCGTGGTTGGT-3′	350	Sei et al. [23]
C23OF C23OR	5′-AAGAGGCATGGGGGCGCACCGGTTCGA-3′ 5′-TCACCAGCAAACACCTCGTTGCGGTTGCC-3′	900	Sei et al. [23]

**Table 2:** Some PAHs catabolic genes detected in ASU-06 using specific primers for each gene.

Gene	Strain	
	*S. koreensis *	Expected band (bp)
*AlkB *	+	100
*AlkB1 *	+	550
*NahAc *	+	487
*PAH-RHD* *α*	−	292
*C12O *	+	350
*C23O *	+	900

**Table 3:** The compounds identified through GC-MS analysis and their mass fragmentation pattern formed by *Sphingomonas koreensis* after 15 days of incubation in MBS containing (100 mg/L) naphthalene, phenanthrene, and pyrene, separately.

Number	Metabolites	RT (min)	*m/z* (%)
1 (cont.)	Naphthalene	25.56	128 (M^+^, 100), 102 (10)
2 (6 d)	1-Methoxynaphthalene	17.37	197 (M^+^, 3), 153 (65), 128 (100), 102 (6)
3 (15 d)	1-Hydroxy-2-naphthoate	23.63	237 (M^+^, 18), 188 (100), 141 (39), 128 (12), 107 (28), 86 (20), 53 (71)
4 (15 d)	2-Hydroxy 4-methoxy cinnamate	55.17	239 (M^+^, 10), 178 (100), 161 (60), 133 (30), 55 (17)
5 (6 d)	Salicylate	28.45	179 (M^+^, 8), 137 (100), 122 (12), 97 (20), 57 (40)
6 (6, 15 d)	Phthalate	51.84	279 (M^+^, 10), 180 (2), 167 (42), 149 (100), 71 (12), 57 (20),
7 (15 d)	Phthalate 3,4-dihydrodiol	47.24	267 (M^+^, 2), 202 (100), 174 (3), 149 (36), 137 (1), 101 (12), 98 (5)
8 (15)	Pyruvate	8.12	101 (M^+^, 5), 89 (70), 73 (5), 71 (100), 56 (53)
1 (cont.)	Phenanthrene	44.59	178 (M^+^, 100), 152 (6), 76 (5)
2	1-Hydroxyphenanthrene	53.71	197 (M^+^, 5), 193 (100), 178 (12), 127 (12), 95 (25), 69 (16), 60 (23)
3	Phthalate	58.97	279 (M^+^, 12), 219 (3), 167 (42), 149 (100), 57 (14)
4	Dihydroxy-cis, cis muconate semialdehyde	48.21	219 (M^+^, 2), 175 (10), 167 (100), 142 (5), 101 (10), 57 (12)
5	2-Hydroxy-4-methoxy cinnamate	55.43	239 (M^+^, 10), 178 (100), 161 (58), 133 (30), 55 (15)
1 (cont.)	Pyrene	38.59	202 (M^+^, 100), 174 (3), 150 (5), 135 (1), 122 (3), 101 (35), 88 (10), 74 (4), 62 (2), 50 (2), 28 (3)
2 (12 d)	1-Hydroxypyrene	39.743	218 (M^+^, 100), 189 (25), 174 (2), 163 (5), 150 (2), 139 (2), 109 (15), 95 (13), 81 (4), 63 (3)
3 (15 d)	4,5-Dihydroxypyrene	43.239	236 (M^+^, 100), 200 (35), 174 (5), 150 (3), 118 (18), 100 (36), 74 (5), 51 (2), 28 (2)
4 (12)	Phenanthrene-4,5-dicarboxylate	47.125	254 (M^+^, 19), 236 (15), 221 (100), 202 (10), 167 (25), 149 (40), 128 (21), 113 (38), 96 (35), 40 (100)
5 (12 d)	3,4-Dihydroxyphenanthrene	38.287	208 (M^+^, 100), 193 (80), 176 (15), 164 (30), 150 (20), 132 (32), 118 (28), 105 (39), 79 (60), 51 (30)
6 (9 d)	1-Hydroxy-2-naphthoate	19.71	188 (M^+^, 100), 146 (5), 119 (30), 105 (55), 91 (73), 78 (10), 65 (15), 51 (3), 41 (5)
7 (15 d)	Benzocoumarin	68.58	224 (M^+^, 15), 207 (50), 191 (10), 163 (15), 147 (13), 120 (80), 92 (48), 63 (25), 44 (100), 16 (83)
8 (12 d)	trans-2-Carboxy-benzalpyruvate	37.426	203 (M^+^, 100), 189 (9), 175 (5), 149 (5), 137 (1), 123 (1), 101 (15), 88 (10), 75 (3), 40 (12)
9 (all d)	Phthalate	46.45	297 (M^+^, 10), 167 (30), 149 (100), 132 (1), 113 (15), 93 (2), 71 (25), 57 (35), 43 (22)
10 (12 d)	Phthalate 3,4-dihydrodiol	37.676	202 (M^+^, 100), 182 (19), 151 (65), 133 (32), 114 (1), 101 (20), 88 (8), 63 (12)
11 (6 d)	3,4-Dihydroxyphthalate	39.7	211 (M^+^, 5), 195 (60), 167 (25), 146 (10), 132 (26), 117 (31), 105 (5), 90 (17), 76 (60), 63 (20), 40 (100)
12 (6 d)	Carboxy-cis, cis-muconate	40.023	183 (M^+^, 15), 168 (25), 155 (20), 141 (10), 124 (21), 111 (21), 97 (20), 85 (25), 71 (20), 57 (55), 40 (100)
13 (15 d)	1-Methoxy, 2-hydroxypyrene	48.32	247 (M^+^, 100), 217 (50), 201 (100), 189 (50), 174 (10), 150 (5), 100 (20), 87 (5)
14 (6 d)	1-Methoxyphenanthrene	38.2	208 (M^+^, 100), 193 (80), 177 (5), 164 (30), 150 (20), 132 (32), 118 (28), 105 (39), 79 (60), 51 (30)

Retention times (RT) and, mass per charge ion (*m/z*), (%) abundancy. Incubation time per days (d).
